# A System of Practical Medicine

**Published:** 1886-04

**Authors:** 


**A System of Practical Medicine.** By American authors. Edited by WM. PEPPER, M. D., LL.D., Provost and Professor of the Theory and Practice of Medicine, and of Clinical Medicine in the University of Pennsylvania, assisted by Louis Starr, M. D., Clinical Professor of Diseases of Children in the Hospital of the University of Pennsylvania. Vol. IV. Philadelphia: Lea Bros. & Co. 1886.

The successive appearance of the volumes of this truly magnificent work has called forth expressions of admiration and satisfaction. The present volume is devoted to the consideration of the diseases af the genito-urinary and cutaneous systems, medical opthalmology and otology. Diseases of the muscular system (though properly belonging in Vol. V., with diseases of the nervous system) have also been placed in this volume. The subjects, myalgia, progressive muscular atrophy, and pseudo-hypertrophic paralysis are treated respectively by Wilson, Tyson, and Mary Putman Jacobi. Duhring and Stellwagen furnish an excellent series of chapters on diseases of the skin. Norris and Strawbridge contribute excellent articles on medical opthalmology and otology The diseases of the genito-urinary system occupy nearly 500 pages of the work, and the names of Delafield, Tyson, Keyes, Gross, Goodell, Gaillard Thomas, Byford and Skeene are sufficient assurance to our readers that the subjects treated receive full justice from the pens of these distinguished authors. The Lea's have done the profession a real service in publishing this great work. They have been fortunate in their editors. As we said in our first notice of the work, there are few men in America better qualified for the editorship of an American system of medicine than Prof. Pepper, and in this work he has added to his reputation. The publishers are famous for the quality of their work; and in print, binding and general "get up," these volumes are beyond criticism.

